# ANNUAL EXPOSURE OF THE SWISS POPULATION FROM MEDICAL IMAGING IN 2018

**DOI:** 10.1093/rpd/ncab012

**Published:** 2021-02-27

**Authors:** Anaïs Viry, Julie Bize, Philipp R Trueb, Barbara Ott, Damien Racine, Francis R Verdun, Régis LeCoultre

**Affiliations:** Institute of Radiation Physics (IRA), Lausanne University Hospital, Lausanne, Switzerland; Institute of Radiation Physics (IRA), Lausanne University Hospital, Lausanne, Switzerland; Radiation Protection Division, Swiss Federal Office of Public Health, Bern, Switzerland; Radiation Protection Division, Swiss Federal Office of Public Health, Bern, Switzerland; Institute of Radiation Physics (IRA), Lausanne University Hospital, Lausanne, Switzerland; Institute of Radiation Physics (IRA), Lausanne University Hospital, Lausanne, Switzerland; University of Health Sciences (HESAV), University of Applied Sciences Western Switzerland (HES-SO), Lausanne, Switzerland

## Abstract

Nationwide surveys on radiation dose to the population from medical imaging are recommended in order to follow trends in population exposure. The goal of the 2018 survey was to investigate the current exposure. The invoice coding information was collected in five university hospitals and large clinics. To improve the estimation of the effective dose delivered in computed tomography (CT), we collected dose data from different Dose Archiving Communication Systems. On average, we found that 1.2 radiological examinations per year and per inhabitant were performed. Dental radiography was the most frequent examination (48% of all the X-ray examinations), followed by conventional radiography (36%) and CT (11%). The average annual effective dose was estimated to be 1.48 mSv per inhabitant, with CT representing 64% of that dose. Our results show that the exposure of the Swiss population from medical imaging has remained stable since 2013, despite a 15% increase in the number of CT examinations.

## INTRODUCTION

In medicine, the use of ionizing radiation for diagnostic and therapeutic procedures plays a major role in patient care. It accounts for almost all sources of man-made exposure and is the second largest contributor to overall population exposure worldwide^(1)^. Monitoring the exposure of the population from medical imaging is a legal obligation both at the European level (EURATOM 2013/59) and at the Swiss level^(2,3)^. Monitoring makes it possible to follow the evolution of the average annual effective dose per inhabitant, to compare with the practice of other countries and to prioritize actions in radiation protection. Indeed, over the last 30 years, the number of medical imaging examinations has considerably increased for diagnostic and therapeutic procedures. In particular, >50 000 computed tomography (CT) devices have been documented to exist around the world, and the expansion of CT concerns all western countries^(4)^. The extensive use of CT comes from its major role in diagnostics (alone or coupled with nuclear medicine devices) and screening (only in a few countries), for its role in minimally invasive procedures (percutaneous abscess drainage, percutaneous biopsy, etc.). The last survey carried out in Switzerland was in 2013 and it reported the annual average effective dose per inhabitant to be 1.38 mSv without the nuclear medicine contribution^(5)^. The highest contribution to the collective radiation dose was from CT examinations (70.4%), despite its relative infrequency (9.6%) compared with other X-ray imaging modalities. The frequency of CT examinations had continuously increased from 2008 to 2013, leading to a 17% increase in the average annual effective dose per inhabitant.

The aim of this study was to estimate the Swiss population’s exposure from medical X-rays in 2018 and to do this by addressing a survey to all medical health care providers. The contribution of the different X-ray imaging modalities (radiography, mammography, dental radiology, CT, interventional radiology and the nuclear medicine) was estimated in order to determine the changes that occurred in 5 years and compare the Swiss practice with other European countries. The previous survey dedicated to the practice of nuclear medicine in Switzerland was performed in 2010 and had no link with the surveys performed in diagnostic radiology. With the general use of hybrid imaging^(6)^ (Single-photon emission computed tomography (SPECT-CT) or Positron-emission tomagraphy (PET-CT)), it appeared important to simultaneously perform a survey to assess the practice of diagnostic radiology together with the practice of nuclear medicine. In comparison with the previous survey, an effort has been made to improve the collection of data for CT in terms of frequency and effective dose. The estimation of the effective dose vector was performed using real dose data from CT examinations since it is the largest dose component in such surveys.

## MATERIAL AND METHODS

In order to carry out such a survey, it is necessary to determine, on the one hand, the frequency of X-ray examinations and, on the other hand, the average effective dose delivered per X-ray modality. The term ‘examination’ was defined in order to correctly extract the frequency of examinations. It represents one or more exposures of an anatomical region or organ, using a single radiological modality, to answer a specific clinical question, during a single visit to a department, hospital or clinic.

### Establishment of the frequency of X-ray examinations

In order to estimate the frequency of X-ray examinations, a hybrid methodology was used combining various sources. First, the Swiss medical billing system (TARMED) was used to extract the number of X-ray examinations in the university hospitals and large clinics for CT, conventional radiology, interventional radiology and diagnostic mammography. In addition, we studied existing reports published by specific Swiss medical societies to get the frequency of the mammography screening and interventional cardiology practices^(7,8)^. Finally, all Swiss owners of radiological installations, not contacted for their TARMED codes, were questioned with web or paper formularies. These were the following imaging modalities: conventional radiography, CT, diagnostic mammography, dental X-ray and nuclear medicine.

The methodology used to extract the required information from the TARMED system was previously described by LeCoultre in a pilot study^(9)^ and in the previous survey^(5)^. The data was processed using the METAXA computer code (METAdata eXtraction and Analysis). To ensure the reliability of the data, we systematically verified these algorithms by comparing the data obtained from the analysis of TARMED codes with the data provided by the radiological information system (RIS) of a few hospitals.

### Extrapolation

As the frequencies of all radiological practices could not be determined, we needed to extrapolate some data to estimate the frequencies at the national level. Indeed, getting access to the TARMED data was often difficult since they are considered politically sensitive by numerous hospitals and clinics. Fortunately, one Swiss canton (representing 10.7% of the population) provided us a complete set of TARMED data for CT, conventional radiology and diagnostic mammography. To extrapolate our data at national level, we could have simply divided our set of detailed data by 0.107 since we had a coverage for 10.7% of the whole population. Nevertheless, we choose to make the hypothesis that the indication of radiological examination is quite homogeneous within the country using a common ratio of the X-ray imaging examinations to the general medical examinations. Using this approach, the factor used for extrapolation was 0.101 (instead of 0.107 when using a simple population scaling). To complete and strengthen our TARMED data set, we also used data provided by either medical societies (for cardiology and mammography screening, for example) or the data collected on web-based forms (for dental radiology, diagnostic mammography and nuclear medicine, for example). The response rate was then used to extrapolate the data at the national level, equal to 18.1% for diagnostic mammography, 29.1% for X-ray dental practice (excluded cone-beam computed tomography (CBCT)), 27% for CBCT and 94.1% for nuclear medicine.

### Establishment of the effective dose vector for each modality

For each radiological modality, a list of the most common types of anatomical regions investigated was defined. For each type of localization and each modality, an effective dose was estimated using different sources: (1) national diagnostic reference level (DRL) data, (2) published data or (3) real dose data. The effective dose vector per modality was then calculated by combining the frequency of each localization with its effective dose.

The effective dose vector for radiography, fluoroscopy and dental radiology was derived from the various national dose surveys carried out over the last 10 years.

The contribution of CT to the collective dose being quite important, we decided to use an innovative approach using real patient dose extracted from Dose Archiving and Communication System (DACS) data. We extracted the median dose length product (DLP) for the most common CT examinations (Table 1) from different DACS systems during 2018 and 2019. These data were processed according to their origin: a set of DLPs related to the practice of university hospitals and a set of DLPs related to the practice of private radiology centers and small regional hospitals were extracted since their practice appeared to be slightly different. To get effective doses, we used the conversion factors published by Deak *et al*.^(10)^. The effective dose vector for CT was then calculated by combining the frequency of each localization, obtained from TARMED data, with its effective dose.

**Table 1:** Contribution in frequency and dose of the different CT procedures.

Anatomical regions	Frequency for 1000 inhabitants	Frequency (%)	Median effective dose (mSv)	Contribution in terms of dose (mSv)	Contribution in terms of dose (%)
Head	19	14.1	2.36	0.329	4.7
Face, sinus	5.3	3.9	2.36	0.091	1.3
Dental CT	0.2	0.21	0.6	0.001	0.01
Neck	7.7	5.7	2.1	0.112	1.7
Chest	15.9	11.8	3.8^a^	0.447	6.3
Abdomen	23.9	17.7	10.5	1.85	26.2
Chest and abdomen	22.1	16.4	12.1	1.974	27.9
Pelvis	10.6	7.9	7.9	0.617	8.8
Spine	15.9	11.8	10.7	1.261	17.7
Shoulder	1.1	0.80	5.8	0.044	0.70
Elbow	0.6	0.40	3.2	0.004	0.20
Wrist/hand	1.3	1.0	1.9	0.019	0.30
Hip	3.6	2.6	11	0.273	4.1
Knee	4.3	3.2	2.7	0.085	1.2
Ankle/foot	3.4	2.5	0.06	0.002	0.02
All examinations	135	100.00	Dose vector	7.1	100.00

The median effective dose was estimated by computing the mean for each procedure between the university practice and the non-university practice.

^a^Large differences can be noted between university hospitals (2.9 mSv) and private radiology centers or regional hospitals (4.7 mSv).

For the nuclear medicine practice, we used the most recent Swiss DRL survey established in 2017 to extract the effective dose coming from radiopharmaceutical products, and we then used various publications for the dose coming from non-referenced radiopharmaceutical products^(11–13)^. The median DLP for CT examinations associated with SPECT or PET examinations was extracted from the publication of Lima *et al*.^(14)^, distinguishing the CT used to obtain the attenuation correction map from the CT used to obtain additional diagnostic information.

### Uncertainties associated with estimating the average effective dose per inhabitant

The average effective dose per inhabitant was calculated by combining the frequency of each modality with its average effective dose vector. Uncertainties came from estimating the frequency of radiological examinations and the effective dose for each modality. Due to the use of various sources with an insufficient number of details, it was not possible to perform a complete analysis of uncertainties. However, uncertainties were subjectively described for the estimation of the frequency of X-ray examinations based on the comparison between TARMED data and RIS data (Table 2). For the estimation of the effective dose in CT, we evaluated the uncertainties using the DLP distribution for each CT procedure extracted from the DACS.

**Table 2:** Data verification between TARMED data and RIS data.

Frequency for 1000 inhabitants	TARMED data	RIS data	Difference (%)
X-ray radiography	439	421	4.10
CT	135	109	19.26
Diagnostic mammography	21.1	19.1	9.48
Total	595.1	549.1	7.73

## RESULTS

The following results present the frequency of X-ray techniques for 1000 inhabitants, the average effective dose per modality and the average effective dose per inhabitant.

### Average effective dose per inhabitant over the various radiological modalities

**Figure 1:**
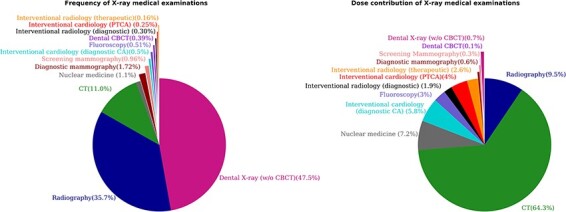
Distribution in percentage of the frequency and dose contribution of X-ray medical examinations.

According to the data presented in Table 3, the average dose per inhabitant due to X-ray imaging is estimated to be 1.49 mSv (1.38 when excluding nuclear medicine). The average number of examinations per inhabitant is 1.2. Figure 1 shows the contribution in terms of frequency and dose for each modality. The most common radiological examination was the dental X-ray, with a frequency of 47.89%, followed by conventional radiography with a frequency of 35.7%. The highest contribution to the collective radiation dose was from CT examinations (64.3%), despite its relative infrequency (11%) compared with other X-ray imaging modalities. The second contributor was conventional radiography (9.5%), followed by nuclear medicine (7.2%).

**Table 3.** Contribution in frequency and dose of the different modalities for the year 2018

X-ray medical imaging modalities	Frequency for 1000 inhabitants	Effective dose vector (mSv)	Dose/inhabitant (mSv)
Conventional radiography	439	0.32	0.14
Diagnostic mammography	21.1	0.36	0.008
Screening mammography	11.8	0.36	0.004
Dental radiography (without CBCT)	584	0.02	0.012
CBCT	4.7	0.2	0.001
CT	135	7.08	0.956
Conventional radioscopy	5.5	8	0.044
Coronary angioplasty (CA)	6.2	14	0.086
Other diagnostic interventional radiological procedures	3.7	8	0.029
Percutaneous transluminal coronary angioplasty (PTCA)	3	20	0.06
Other therapeutic interventional radiological procedures	1.9	20	0.038
Total X-ray medical imaging without nuclear medicine	1215.8	—	1.378
Nuclear medicine	13.3	8.04	0.107
Total X-ray medical imaging with nuclear medicine	1229.1	—	1.485

### Frequency and effective dose for CT examinations

Table 1 presents the frequency of CT examinations and their effective dose contribution. As expected, the most common CT examination was the abdominal CT and the combined examination of chest and abdomen, with a frequency of 17.7 and 16.4%, respectively. Verifying the data enabled us to assess the uncertainty associated with the estimation of the frequency of CT examinations at about 20% (Table 2). The median effective dose was equal to 10.5 and 12.1 mSv, respectively, representing the two largest contributors to the CT effective dose for the population.

**Table 4:** Evolution of the frequency and dose contribution of the different modalities (except nuclear medicine) between 2013 and 2018.

Modality	Frequency for 1000 inhabitants		Effective dose per inhabitant (mSv)	
	2013	2018	2013	2018
Conventional radiography	473	439	0.151	0.140
Diagnostic mammography	20	21.1	0.007	0.008
Screening mammography	11	11.8	0.004	0.004
Dental radiology (without CBCT)	572	584	0.011	0.012
CBCT	6	4.7	0.001	0.001
CT	117	135	1.000	0.956
Conventional radioscopy	7	5.5	0.059	0.044
Coronary angioplasty (CA)	6	6.2	0.080	0.086
Other diagnostic interventional radiological procedures	2	3.7	0.017	0.029
Percutaneous transluminal coronary angioplasty (PTCA)	3	3.0	0.054	0.060
Other therapeutic interventional radiological procedures	2	1.9	0.034	0.038
Total X-ray medical imaging (without nuclear medicine)	1219	1215.8	1.42	1.38

The average effective dose for all CT examinations was equal to 7.1 +/− 0.5 mSv, taking into account all dose data for the CT effective dose and all TARMED data for the frequency. The analysis of dose data from DACS showed a variation in practice between the university hospitals and the private or regional hospitals. Particularly, the effective dose of chest CT examinations was 40% lower in comparison with private or regional hospitals. Taking the various practices into account, the average effective dose could be estimated at 6.73 mSv using dose data from university hospitals only or 7.5 mSv using dose data from non-university hospitals. This difference shows that the uncertainties associated with the estimation of the CT effective dose was around 8% (ratio between the standard deviation and the average effective dose).

### Changes within 5 years

According to our results, between 2013 and 2018, there was a decrease in the number of conventional radiography and conventional radioscopy sessions by 7 and 21%, respectively (Table 4). CT still delivered 0.96 mSv per inhabitant in 2018, although its average dose per exam had decreased by 17% over the 5-year period. The frequency of CT examinations increased by 15%. We also observed a 19% increase in interventional radioscopy sessions for diagnostic purposes over the same 5 years. All the other radiological modalities remained relatively stable, both in terms of frequency and in terms of dose. There was a decrease of 0.3% in the number of X-ray examinations per 1000 inhabitants, as well as a decrease of 3% of the exposure of the population in the medical sector, excluding nuclear medicine. Despite the uncertainties associated with this estimation, the exposure of the population from medical imaging appears to be stable since 2013.

## DISCUSSION

This analysis of frequency and effective dose shows that the noticeable increase in patient exposure from medical imaging between 2008 and 2013 has reached a plateau. The exception to this trend is the continuous increase in the number of CT examinations. However, the CT effective dose decreased during this 5-year period as the result of a joint effort between manufacturers, radiologists, radiographers and medical physicists, all dedicated to the optimization of clinical protocols.

The major strength of our study was the methodology used to collect the frequency of X-ray examinations using TARMED codes, a method identical to that used in the 2013 survey. This enabled us to correctly analyze the trend over 5 years. Moreover, almost all Swiss practitioners were contacted, and even if the answer rate is commonly rather low with this type of survey, it was possible to gather broad data on radiological practices, for both hospitals (university or regionals) as well as private imaging centers. The major improvement between this study and the previous survey was our estimation of the frequency of CT examinations and the associated effective dose. The number of CT invoice codes were first corrected in order to highlight the number of sessions combining two or more similar regions of interest, with the aim of particularly differentiating thoraco-abdominal CTs, chest CTs alone and abdominal CTs alone. Indeed, the number of CT examinations that combine several anatomical regions is an increasingly frequent practice in the clinical routine, and this had to be taken into consideration in the estimation of CT frequency and associated effective dose. Moreover, the analysis of different DACS systems, from university hospitals and regional hospitals, made it possible to conduct dose estimations that reflect real daily clinical routine while considering differences of practice.

In our comparison with other countries, the 2018 Swiss values were close to the values published in France in 2012 and 2017^(15,16)^, in Germany in 2014^(17)^, in Austria in 2015^(18)^ and in the USA for 2016^(19)^. Like Switzerland, the frequencies of the radiography, mammography and dental radiology also remain stable in these countries. Germany has noted an increase of 40% in the number of CT examinations in 7 years; this is comparable with the 34% increase in Switzerland between 2008 and 2018^(20)^.

The main limitation of our study was our need to make various assumptions in order to extrapolate the data at the national level from the data coming from different sources. The evaluation of uncertainties is another important limitation. Nevertheless, we made an effort to evaluate the uncertainties coming from CT examinations since they represent the major source of radiation exposure.

## CONCLUSION

In conclusion, our study showed that exposure of the Swiss population coming from medical imaging was stable, despite an increasing tendency in the number of CT examinations. Regardless of this stabilization, radiation protection efforts should be continued; first, with the justification of every examination, particularly in CT, and second, through the optimization of the various radiological procedures. The implementation of clinical audit focusing on justification is an adequate instrument to address the first point.
